# Navigating the Intersection of Autism and Gender Fluidity: A Case study of psychotherapy in a Neurodivergent Adult

**DOI:** 10.1192/j.eurpsy.2025.2157

**Published:** 2025-08-26

**Authors:** A. Vuk

**Affiliations:** Department of integrative psychiatry, University psychiatric hospital Sveti Ivan, Zagreb, Croatia

## Abstract

**Introduction:**

Autism Spectrum Disorder (ASD) is a developmental condition that modifies how a person perceives their surroundings, communicates and interacts with others. Autistic individuals often experience executive dysfunction, impaired social cognition, likewise differences in perception and information processing that can make it challenging to describe their internal sense of gender and gender-related needs. This case report presents the therapeutic journey of a young man diagnosed with Asperger Syndrome who entered dynamic supportive psychotherapy to address social and emotional challenges, likewise identity issues.

**Objectives:**

This poster aims to explore the clinical complexities arising from the presence of gender neurodivergence in individuals with ASD and its impact on their presentation, assessment and management. It seeks to highlight the unique challenges faced by autistic individuals in navigating gender identity, emphasizing the need for tailored clinical approaches.

**Methods:**

A descriptive case report of a patient with ASD and gender neurodivergence, drawing on clinical records, patient interviews and psychotherapy sessions, psychological assessment and a non-systematic review of relevant literature.

**Results:**

A case report presentation of a 29-year-old man diagnosed with Asperger syndrome during adolescence. Due to adaptation difficulties and anxiety he started seeing a child’s psychiatrist and then transferred to an adult setting. He had intermittent visits to a community mental health service initially and, over the last two years, attended biweekly psychotherapy sessions at our hospital. Early psychological assessments revealed challenges with social interactions, confused and bizarre thinking patterns, identity diffusion, and a tendency for ruminative self-reflection. Treatment was interrupted at the end of 2023 due to a psychotic episode with dissociative phenomena, leading to the introduction of antipsychotics. After stabilization, psychotherapy resumed. During the course of therapy, conversations revealed a complex narrative around gender identity, including reflections on multiple genders and gender fluidity.

**Conclusions:**

This case highlights the intersection of neurodiversity and gender identity, illustrating how individuals on the autism spectrum may experience and navigate gender in unique ways. The therapeutic process explored gender fluidity as an evolving self-concept, emphasizing the importance of inclusive approaches in psychotherapy. The report underscores the need for mental health professionals to be attuned to diverse expressions of gender identity within neurodivergent populations and advocates for flexible therapeutic frameworks that accommodate both neurodiversity and gender diversity.

**Disclosure of Interest:**

None Declared

